# Pediatric rheumatic diseases and vaccinations: a promising alliance

**DOI:** 10.3389/fped.2025.1672792

**Published:** 2025-11-27

**Authors:** Giovanni Corsello, Carla Gilotta, Clotilde Genesia Alizzi, Guglielmo Francesco Benfratello, Maria Cristina Maggio

**Affiliations:** 1 PROMISE: Promozione della Salute, Materno-Infantile, di Medicina Interna e Specialistica di Eccellenza “G. D'Alessandro”, University of Palermo, Palermo, Italy; 2 UOC of Pediatria Generale, Children Hospital “G. Di Cristina”, ARNAS Civico, Palermo, Italy; 3 Department of Physics, University of Pisa, Pisa, Italy

**Keywords:** children, rheumatic diseases, flu vaccination, COVID-19 vaccination, disease-modifying antirheumatic drugs

## Abstract

**Background:**

Immunosuppressive drugs, disease-modifying antirheumatic drugs, as methotrexate, glucocorticoids and biological agents can limit the immune response to vaccines and, in some cases, contraindicate their administration. Non-live vaccines are safe also for immunosuppressed paediatric patients. Seroprotection is maintained in children with rheumatic diseases or autoinflammatory diseases (AID) undergoing vaccinations on immunosuppression, except for those treated with high-dose glucocorticoids and B-cell depleting drugs.

**Methods:**

We analyzed in a retrospective observational study 107 patients (36 males; 71 females) with rheumatological diseases and AID. Median age was 7 ± 4 years. Patients were divided into four groups, based on the principal diagnosis: group 1) included 58 patients with juvenile idiopathic arthritis (JIA); group 2) included 30 patients with monogenic AID; group 3) included 14 patients with other rheumatological diseases (SLE, recurrent uveitis, vasculitis, Behçet's disease) and group 4) included 5 patients with MIS-C, Kawasaki disease. We assessed adherence to mandatory vaccinations, as well as seasonal influenza and Coronavirus disease 2019 (COVID-19) vaccinations.

**Results:**

The vaccination campaign did not obtain the expected results. All the patients, except for two children, showed a good adherence to mandatory vaccinations. The two unvaccinated children were affected by JIA, they were treated with methotrexate (one) and methotrexate plus adalimumab (the other) and did not undergo vaccination because their parents feared a recurrence of the disease. After an appropriate motivational interview with the parents, both children completed the vaccination schedule required for their age. Among the patients, 14% were vaccinated against both influenza and COVID-19; 32% only against COVID-19 and 15% only against seasonal flu; conversely, 39% were not vaccinated nor against flu or against COVID-19.

**Conclusions:**

It is necessary to support families in the decision to join the vaccination campaigns, with exhaustive information about the benefits of vaccinations also as promoters of a better quality of life.

## 1 Introduction

Pediatric rheumatic diseases and autoinflammatory diseases (AID) are chronic conditions, often loaded by an increased risk of infections, comorbidities and a reduced quality of life. Immunosuppressive drugs, disease-modifying antirheumatic drugs (cDMARDs), as methotrexate, glucocorticoids and biological drugs can limit the immune response to vaccines and, also, contraindicate their administration, particularly in the case of live vaccines. Recent studies, however, documented the safety of the latter in patients taking low doses of glucocorticoids and methotrexate, highlighting the appropriate timing for vaccine dose. Guidelines propose to guarantee a variable interval, based on the type of treatment, for the administration of inactive vaccines to promote a better immunological response and to guarantee clinical remission, the optimal condition to carry out the vaccine.

Safe vaccination programs suggest that vaccines have no severe adverse effects, do not worsen the underlying disease, also considering the appropriate evaluation of disease activity by specific scores (1–4), and do not begin infections in case of live-attenuated vaccines.

Treatment with glucocorticoids can induce immune suppression by several mechanisms of action. Glucocorticoids passively cross the cellular membrane and bind to the specific intracellular receptor, generating a complex that arrives at the nucleus and binds DNA (5).

Anti-inflammatory action of glucocorticoids depends on the inhibition of the synthesis of several inflammatory cytokines by blocking the function of various transcription factors, such as nuclear factor kappa B (NF-kB) and activator protein-1 (AP-1), which are needed for proinflammatory mediators' transcription. Besides, glucocorticoids anti-inflammatory effects depend on the block of the action of proinflammatory genes, such as interleukin (IL)-1-alpha and IL-1-beta genes (6). Glucocorticoids decrease peripheral lymphocytes, monocytes, basophils, and eosinophils number. Glucocorticoids reduce neutrophil adherence to the vascular endothelium. Glucocorticoids inhibit the function of macrophages and other antigen-presenting cells by limiting chemotaxis, and thus bactericidal activity, by the inhibition of the release of cytokines such as tumor necrosis factor and IL-1. Glucocorticoids induce leukocytosis, reducing leukocytes' adhesion to the vascular endothelium and the leaving from the circulation. These actions are linked to the impaired migration into sites of infection and tissue injury. Neutrophilia also results from an increased liberation of mature polymorphonuclear leukocytes from the bone marrow into circulation, associated with decreased apoptosis.

Furthermore, immune suppression is dose-dependent, as demonstrated *in vitro* and *in vivo* by several studies (7–12).

Children are considered immunosuppressed if treated with a prednisolone dose ≥0.5 mg/kg/day during ≥2 weeks. Furthermore, children are considered immunosuppressed when treated with cDMARDs at the following dosages: methotrexate ≥15 mg/m^2^/week or ≥25 mg/week, mycophenolate mofetil ≥30 mg/kg/day or >1,000 mg/day, cyclosporine >2.5 mg/kg/day, azathioprine ≥3 mg/kg/day, cyclophosphamide orally >2.0 mg/kg/day, leflunomide ≥0.5 mg/kg/day, tacrolimus >1.5 mg/day. All patients are considered immunosuppressed when using biological DMARDs (bDMARDs), or targeted synthetic DMARDs (tsDMARDs). Lastly, patients are considered immunosuppressed when using a combination of the aforesaid drugs, at any dosage (13).

Recent studies proving the safety of live-attenuated vaccines and original studies evidencing the immunogenicity of vaccinations in the era of DMARDS and biological drugs in pediatric patients with autoimmune, rheumatic diseases and AID, contributed to update the European Alliance of Associations of Rheumatologists (EULAR) recommendations (13). The recommendations from EULAR and Paediatric Rheumatology European Society (PReS) for vaccination of paediatric patients with rheumatic diseases and AID support non-live vaccines for these patients. These types of vaccines can also be administered to immunosuppressed children. Generally, seroprotection is preserved and maintained in immunosuppressed children, except in children treated with high-dose glucocorticoids and B-cell depleting drugs. Hence, live-attenuated vaccines should be avoided in immunosuppressed patients. Nevertheless, the measles–mumps–rubella booster and varicella zoster virus vaccine are safe for immunosuppressed patients under specific conditions. Furthermore, the non-live seasonal influenza vaccination should be supported for children with rheumatic diseases and AID (13).

The National Immunisation Programmes (NIP) should be reviewed yearly by paediatric rheumatologists. In patients for whom this is possible, vaccinations should be completed before starting immunosuppressive therapy, but required treatment should never be delayed. For patients receiving immunosuppressive therapy, an additional booster dose is suggested, without any modification of the therapeutic scheme, at least 3 months later.

It is still mostly unknown whether children with high disease activity may be safely and effectively vaccinated. It is recommended to vaccinate during the inactive disease when it is possible, but there is no absolute contraindication to vaccinate during high disease activity. The option could be guided by the epidemic reality.

The objective of this study is to analyze the vaccinations coverage for influenza and COVID-19 of our patients with rheumatic diseases or AID. Moreover, we supported the importance of vaccinations for patients with rheumatic diseases or AID and we promoted anti-flu and anti-COVID-19 vaccinations.

## 2 Methods

We analyzed in a single-center retrospective observational study a series of 107 patients (36 males; 71 females; medium age: 7 ± 4 years) with rheumatological diseases and AID, followed at the “Pediatric Rheumatology” unit of the P.O. “G. Di Cristina”, ARNAS of Palermo.

We included in the study all the patients followed in our unit with an active follow-up and who responded to our questions about vaccinations. The recall bias is limited, because we verified the referred data with the official vaccine card of each patient. In fact, an essential element for inclusion in the study was the access to the patient's vaccinations records. Vaccination records were obtained from patients' vaccination cards, compiled and updated by vaccination centres, based on the registration of vaccinations in the national vaccination register.

We divided them into four groups, based on the principal diagnosis and we evaluated their adherence to the seasonal flu and COVID-19 vaccinations.

The four groups were:

Group 1) includes 58 patients (16 males, 42 females; medium age: 13.3 years) with juvenile idiopathic arthritis (JIA). M ± SDS disease duration was 6.9 ± 3.9; M ± SDS treatment duration was 6.6 ± 3.8 years.
Group 2) includes 30 patients (15 males, 15 females; medium age: 13.4 years) with monogenic AID (e.g., familial mediterranean fever (FMF), tumor necrosis factor receptor-associated periodic syndrome (TRAPS), mevalonate kinase deficiency (MVK), cryopyrin-associated periodic syndromes (CAPS). M ± SDS disease duration was 9.5 ± 5.3 years; M ± SDS treatment duration was 9.5 ± 4.3 years.
Group 3) includes 14 patients (3 males, 11 females; medium age: 14 years) with other rheumatological diseases [e.g., systemic erythematous lupus (SLE), recurrent uveitis, vasculitis, Behçet's disease]. M ± SDS disease duration was 5.2 ± 3.2 years; M ± SDS treatment duration was 5 ± 3.5 years.
Group 4) includes 5 patients (2 males, 3 females; medium age: 7.5 years) with multisystem inflammatory syndrome in children (MIS-C), Kawasaki disease. M ± SDS disease duration was 2.8 ± 1.1 years; M ± SDS treatment duration was 2.6 ± 1.2 years.

### 2.1 Statistical analysis

We used Excel to perform the statistical analysis. We used the chi-squared test for contingency tables (groups of patients, vaccines; therapy, vaccines). The statistically significant result is considered a value *p* < 0.05. For the multivariate analysis we used the statsmodels.api Python library to implement the logistic regression.

## 3 Results

All the patients, all patients, except two children, showed a good adherence to mandatory vaccinations. The two unvaccinated children were affected by JIA and treated with methotrexate (one) and methotrexate plus adalimumab (the other). The two children had not been vaccinated because their parents feared that vaccination would cause a recurrence of the disease. After an appropriate motivational interview with the parents, both children completed the vaccination schedule required for their age.

However, despite the continuous solicitation to vaccination proposed to all the parents and the patients, the vaccination campaign did not obtain the expected results. In fact, 15/107 (14%) of patients were vaccinated against both influenza and COVID-19; 34/107 (32%) of patients were vaccinated only against COVID-19; 16/107 (15%) of patients were vaccinated only against seasonal flu and 42/107 (39%) of patients were not vaccinated nor against flu or against COVID-19 (Table 1).

**Table 1:** Percentage of patients vaccinated for influenza and/or COVID-19 in the four groups.

Group of patients, based on the principal diagnosis	Patients vaccinated only for influenza	Patients vaccinated only for COVID-19	Patients vaccinated for influenza and COVID-19	All patients vaccinated for influenza	All patients vaccinated for COVID-19
Group 1	9/58 (15.5%)	16/58 (27.6%)	8/58 (13.8%)	17/58 (29.3%)	24/58 (41.4%)
Group 2	5/30 (16.7%)	14/30 (47.7%)	3/30 (10%)	8/30 (26.7%)	17/30 (56.7%)
Group 3	2/14 (14.3%)	4/14 (28.6%)	4/14 (28.6%)	6/14 (42.9%)	8/14 (57.1%)
Group 4	0/5 (0%)	0/5 (0%)	0/5 (0%)	0/5 (0%)	0/5 (0%)

Patients of group 4 showed low compliance with both vaccinations. Between all the children of the four groups and especially of group 4, some patients were not vaccinated against COVID-19 because they contracted the infection.

Patients in group 1 and group 2 have joined the anti- influenza and anti-COVID-19 combined vaccination in a low percentage because the fear of a relapse of the disease has influenced the parents’ choice to vaccinate their children.

However, the difference of percentage of vaccinated children between groups 1, 2 and 3 did not reach statistical significance.

The association between the flu and the COVID-19 vaccinations was measured using the phi coefficient and then computing the chi-square value. In the two groups with a higher sample number (group 1 and group 2) no association was found, with *p*-values of 0.74 (*φ* = 0.04) and 0.20 (*φ* = 0.23) respectively. These associations were measured in the same way for the different drugs employed to treat the diseases included in group 1 and 2. Treatment considered were: methotrexate, canakinumab and no actual treatment for group 1; colchicine, canakinumab and no treatment for group 2. In all cases, no statistically significant association was found, with *p*-values >0.05 (Table 2).

**Table 2:** The association between the flu and the COVID-19 vaccinations and treatment.

Group 1	*p*-Value	phi
Methotrexate	0.6	0.17
Canakinumab	0.26	0.25
Anti-TNFalfa	0.64	0.65
No immunosuppressive drugs	0.20	0.41
Group 2		
Colchicine	0.69	0.14
Canakinumab	0.09	0.42
No immunosuppressive drugs	0.27	0.45

Logistic regression was used to analyze the relationship between age at the diagnosis and one of the two vaccinations under study (flu or COVID-19 vaccination). Logistic regression was conducted separately in the two biggest groups (group 1 and group 2).

While no significant dependence of one vaccination on the other has been found, in some cases a significant dependence on the age at the diagnosis was detected. In particular, the odds of the flu vaccination increased by 32% [95% CI (1.12, 1.56)] for each year before the JIA diagnosis.

Furthermore, the odds of the anti-COVID-19 vaccination decreased by 26% [95% CI (0.55, 0.99)] for each year before the AID diagnosis.

The same analysis conducted on the other vaccination showed no significant dependencies on the age and the other vaccination in both groups (Table 3).

**Table 3:** Relationship between age at the diagnosis and one of the two vaccinations under study (the last two columns indicate the lower and upper limits of the 95% confidence interval).

Diagnosis	Dependent variable	Independent variable	*p*-Value	Odds ratio	[0.025, 0.975]
JIA	Flu vaccination	COVID-19 vaccination	0.746	1.242	0,335, 4.608
JIA	Flu vaccination	age	0.001	1.322	1.122, 1.559
JIA	COVID-19 vaccination	Flu vaccination	0.732	1.256	0.340, 4.644
JIA	COVID-19 vaccination	age	0.855	1.013	0.878, 1.169
Monogenic AID	Flu vaccination	COVID-19 vaccination	0.404	0.451	0.070, 2.923
Monogenic AID	Flu vaccination	age	0.308	1.160	0.871, 1.544
Monogenic AID	COVID-19 vaccination	Flu vaccination	0.424	0.467	0.072, 3.019
Monogenic AID	COVID-19 vaccination	age	0.041	0.738	0.552, 0.987

Among patients receiving biological drugs, 6 children were treated with anti-TNF-alfa biological drugs in association with methotrexate; 7 children were treated with anakinra (anti-IL-1 receptor antagonist); 38 children were treated with canakinumab (anti-IL-1 beta biological drug); 5 children were treated with roactemra (anti-IL-6 biological drug).

We evaluated the correlation between the drugs taken and the vaccinations carried out.

Patients treated with biological drugs (anti-TNF-alpha, anakinra, canakinumab, roactemra) did not show a statistically significant difference among the different groups of patients, in terms of vaccination against flu and/or COVID-19 (Table 4). However, the difference between the number of vaccinated vs. unvaccinated patients treated with canakinumab showed that vaccinated were more than unvaccinated, with a *p*-value = 0.049. Furthermore, all patients receiving canakinumab underwent mandatory vaccinations. 44.7% of patients treated with canakinumab were vaccinated against influenza, 47.4% were vaccinated against COVID-19. These patients are children with Still's disease (group 1) or AID (group 2).

**Table 4:** Adherence to vaccinations for influenza and/or COVID-19 in correlation with treatment.

Treatment	Patients vaccinated for influenza/total number of pazients receiving the specific treatment	Patients vaccinated for COVID-19/total number of pazients receiving the specific treatment	Patients vaccinated for influenza and COVID-19/total number of pazients receiving the specific treatment	No vaccinations/total number of pazients receiving the specific treatment	*p* Value (vaccinated vs. unvaccinated)
Anakinra	3/7 (42.9%)	0/7 (0%)	0/7 (0%)	4/7 (57%)	0.332
Canakinumab	17/38 (44.7%)	18/38 (47.4%)	6/38 (15.8%)	9/38 (23.7%)	0.049*
Roactemra	2/5 (40%)	3/5 (60%)	1/5 (20%)	1/5 (20%)	0.378
Anti-TNF-alpha	1/4 (25%)	2/4 (50%)	1/4 (25%)	2/4 (50%)	0.66
Anti-TNF-alpha + methotrexate	0/6 (0%)	0/6 (0%)	0/6 (0%)	6/6 (100%)	0.002
Methotrexate	1/11 (9.1%)	3/11 (27.3%)	0/11 (0%)	7/11 (63.6%)	0.098*
Methotrexate alone or + anti-TNF-alpha	1/17 (5.9%)	3/17 (17.6%)	0/17 (0%)	13/17 (76.5%)	0.002*
Colchicine	1/8 (12.5%)	6/8 (75%)	1/8 (12.5%)	2/8 (25%)	0.409
No immunosuppressive drugs	4/21 (19%)	11/21 (52.4%)	4/21 (19%)	10/21 (47.6%)	0.432*

TNF, tumor necrosis factor.

* Groups with a number of patients adequate to perform the chi-square test.

All patients receiving anakinra underwent mandatory vaccinations. 42.9% of patients receiving anakinra were vaccinated against influenza, but none had been vaccinated against COVID-19. These patients are patients with Still's disease (group 1), Kawasaki disease or MIS-C (group 4).

No patients treated with anti-TNF-alpha biological drugs plus methotrexate were vaccinated against flu and against COVID-19. However, the limited number of patients in this subset did not permit to reach statistical significance. Patients treated with methotrexate alone showed a low adherence to both vaccinations, with a difference near the statistical significance.

However, considering all patients treated with methotrexate, alone or in association with anti-TNF-alpha, we showed that adherence to the vaccinations mentioned above was much lower than in other groups of patients treated with other drugs, and this difference reached statistical significance (Table 4).

Nonetheless, mandatory vaccinations were performed by 15/17 (88%) patients treated with methotrexate, except two children with JIA, as previously reported.

No major adverse events were reported during or after vaccinations in all the patients included in the study, excluding short-lived febrile reactions.

## 4 Discussion

It is necessary to support families in the decision to join the vaccination campaigns, with exhaustive information that can make known the benefits of vaccinations also as promoters of a better quality of life.

In recent years an increasing number of studies highlighted the importance of a good lifestyle for patients with chronic diseases, which allows them to integrate in activities, sport, school as their healthy peers (14–17). Treatment with biological drugs help them to reach disease remission and to prevent organ damage and disabilities (16–23). To reach these goals, protection against infections is necessary and vaccines are essential allies for children with rheumatic diseases or AID.

The mistrust and fear which, in most cases, represent the first mover of the failure to carry out the anti- influenza and anti-COVID-19 vaccines, are not currently sustained by studies which support the risks of reactivations or exacerbation of the disease. However, many patients experiment fear for disease flares or complications after vaccinations, such as macrophage activation syndrome in Still's disease or MIS-C (24–27).

All patients treated with canakinumab or anakinra underwent mandatory vaccinations. We observed that patients treated with canakinumab adhered to the influenza vaccination in a percentage comparable to patients treated with anakinra. However, 47.4% of patients treated with canakinumab were vaccinated against COVID-19. This data differs significantly from patients treated with anakinra, as none of them were vaccinated against COVID-19. The difference could not reach statistical significance, for the limited number of children treated with anakinra included in the survey. It is conceivable that the difference in adherence to vaccination against COVID-19 depends in part on the disease from which the children are affected, rather than on the drug used. In fact, the therapeutic target of anakinra and canakinumab and the possible impact on the immune response to vaccines is equivalent for the two drugs.

Commonly, it may be endorsed that children treated with IL-1 blocking drugs can be vaccinated without discontinuing the anti-IL-1 therapy. This recommendation is strongest for patients who have not achieved adequate disease control to avoid disease relapses. Nevertheless, in selected cases, vaccines can be administered seven days after reaching baseline levels of the drug, to avoid a possible reduced response to the vaccine. This measure is especially useful for patients treated with anti-IL-1 drugs with a long half-life, such as canakinumab. The strategy adopted can improve the vaccination coverage of patients, with a view to ensuring that patients with rheumatological diseases or AIDs may present a more severe course of infections such as COVID-19 or influenza (28).

Numerous studies have showed the immunogenicity, the efficacy in preventing the infection and the safety of influenza vaccination. Patients with pediatric AID or rheumatic diseases showed seroprotection rates mostly equivalent to healthy children, except for patients receiving high-dose glucocorticoids (29) and of children with SLE (30), especially when patients had high disease activity (30–33).

Similarly, children treated with bDMARDs (anti-IL6 and anti-TNF alpha) reach seroprotection rates equal to healthy controls, although in patients treated with anti-TNF alpha, specific antibody levels are lower and have a faster decrease (33–36). However, seroconversion alone does not adequately reproduce vaccine immunogenicity in patients with rheumatic diseases receiving immunosuppressive drugs (35).

Non-live vaccines are mostly safe and provide effective seroprotection under IL-1 blockade (28). Studies on the efficacy and safety of inactivated vaccines in children treated with anti-IL-1 bDMARDS, using antibody titers after vaccination, found that influenza vaccination is effective in patients treated with anakinra or canakinumab (37, 38).

The anti-influenza vaccination efficacy has been studied poorly in these pediatric patients. Only two studies reported on influenza infections after vaccination in children with JIA. Both the studies evidenced a lower incidence of infection in unvaccinated children (34, 39).

Patients treated with canakinumab are children with systemic JIA or AID, while patients treated with anakinra are patients with Still's disease (18), Kawasaki disease (40) or MIS-C (27).

Patients with MIS-C did not receive flu and COVID-19 vaccinations because they recently developed the infection of SARS-CoV-2. However, although patients with MIS-C are still followed up at our hospital (26, 27) and are constantly urged and motivated to complete the vaccination card, the parents of the patients included in the study have not had them vaccinated against influenza and COVID-19. Parents had the fear of disease relapse caused by the vaccination as a trigger, and they decided independently, despite the urging for vaccinations by the pediatric rheumatologist and the family pediatrician.

In our study we evidenced that patients treated with methotrexate, alone or in association with anti-TNF-alpha biological drugs, showed a lower adherence to the abovementioned vaccinations than patients of the other groups, treated with other drugs, and this difference is statistically significant. The observation of lower vaccination adherence in patients on methotrexate could have different possible reasons: parents have concern of flare, family paediatrician can fear relapses of the disease and/or a low immunogenicity of vaccinations taken during treatment. In fact, most paediatricians consider methotrexate as an immunosuppressant drug, reducing efficacy of vaccination. The target therapy with biological drugs is considered by family paediatricians and patients' parents to be a therapy with fewer immunosuppressive effects against infections and vaccines.

A topic to consider is that typically, in patients with disease in remission or without recent flare-ups, follow-up visits for children treated with methotrexate and/or anti-TNF-alpha are more spaced, apart than for patients treated with anakinra or canakinumab. This also depends on the disease the patients are suffering from (Still's disease or monogenic AID) and the need to monitor blood tests and clinical parameters, on an outpatient basis, more frequently before administering canakinumab or anakinra.

These direct assessments by the paediatric rheumatologist at our center are supported by constant monitoring of vaccination status and encouragement to adhere to seasonal vaccinations.

However, the limitations of the study are the low number of patients in group 4 (with MIS-C and Kawasaki disease) and the limited number of patients receiving different drugs, especially anakinra, which limits the statistical significance of the correlations. To avoid these bias, we are planning to improve these cohorts, enrolling more patients.

Another limit of the study is that the vaccine immunogenicity and seroprotection has been verified in some patients only for tetanus and hepatitis B.

A study on children with paediatric rheumatic diseases vaccinated with the BNT162b2 mRNA vaccine assessed the humoral response and demonstrated significantly lower median titres of IgG antibodies than those receiving only cDMARDs (41). Other studies evidence safety profile of measles-mumps-rubella booster in children diagnosed with rheumatic diseases receiving bDMARDs and these studies are a strong point that motivates parents and family paediatricians of these patients to adhere to vaccinations (13, 42). As highlighted by many authors, joint decision-making with parents and patients is encouraged in clinical settings (43).

In conclusion, our findings underscore the importance of promoting adequate vaccination coverage, not only for patients, but also for the entire family unit.

In fact, a recent study documented that children with rheumatic diseases show the proportion of incomplete live-attenuated vaccines after diagnosis more than pre-diagnosis, while the proportion of incomplete non-live vaccines before and after diagnosis is similar. The most relevant reasons for missed vaccines are physicians' recommendations, the diagnosis of a rheumatic disease and the drugs prescribed to treat the disease (44).

To avoid these concerns and to improve vaccination efficacy, EULAR/PReS recommendations highlight that vaccines should be, if possible, administered before planned immunosuppression or immune modulating therapies, especially B cell-depleting therapy, to obtain optimal vaccination responses. Though, required immunosuppressive drugs should never be deferred due to vaccination calendar. When immune response is uncertain, specific antibodies of immune response to vaccines antigens should be titled after vaccination and patients can be consequently boostered (13).

The evaluation of the actual seroprotection for each patient should be encouraged, in order to increase adherence to vaccinations.

However, we believe that vaccination strategy should be a joint decision with patients and their caregivers. We need to sensitize families to make a conscious choice and to eliminate misinformation should represent a primary objective to prevent, protect and treat.

## Data Availability

The raw data supporting the conclusions of this article will be made available by the authors, without undue reservation.
